# Fiber Orientation and Strain Rate-Dependent Tensile and Compressive Behavior of Injection Molded Polyamide-6 Reinforced with 20% Short Carbon Fiber

**DOI:** 10.3390/polym15030738

**Published:** 2023-01-31

**Authors:** Joonhee Lee, Hyungyil Lee, Naksoo Kim

**Affiliations:** Department of Mechanical Engineering, Sogang University, Seoul 04107, Republic of Korea

**Keywords:** short fiber-reinforced composites, injection molding, thermoplastic material characterization, numerical simulation, complex geometry

## Abstract

As the interest in short-fiber reinforced polymer (SFRP) composites manufactured by injection molding increases, predicting the failure of SFRP structures becomes important. This study aims to systemize the prediction of failure of SFRP through mechanical property evaluation considering the anisotropy and strain rate dependency. To characterize the mechanical properties of polyamide-6 reinforced with carbon fiber of a weight fraction of 20% (PA6-20CF), tensile and compressive experiments were conducted with different load-applying directions and strain rates. Additionally, the results were discussed in detail by SEM image analysis of the fracture faces of the specimen. FE simulations based on the experimental condition were constructed, and the numerical model coefficients were derived through comparison with experimental results. The coefficients obtained were verified by bending tests of the specimens manufactured from composite cross members fabricated by injection molding. Predicting under static and high strain rate conditions, small errors of about 9.6% and 9.3% were shown, respectively. As a result, it proves that explained procedures allow for better failure prediction and for contribution to the systematization of structural design.

## 1. Introduction

Short fiber reinforced thermoplastic polymers are largely utilized in the industry due to their versatility, recyclability, and low cost and cycle time related to the injection molding process [1]. For these reasons, they have been largely employed in the automotive industry, whose fuel efficiency and emission reduction regulations have been strengthening in recent years. Among the recent regulations, the European one concerning CO2 emission poses a serious challenge to emission reduction (< 95 g/km within 2020). Thus, several automotive manufacturers are researching the direction of replacing aluminum and steel components with reinforced thermoplastic ones because of the considerable weight reduction [2]. In order to properly apply fiber-reinforced thermo-plastic materials to high-performance structural components, the local material properties resulting from the material flow during the thermoplastic injection molding (TIM) process should be properly accounted for. Hence, in the literature, several authors focused their attention on estimating the variation of the mechanical properties of short fiber reinforced (SFR) material, as summarized hereafter.

The effect of the anisotropy developing in injection-molded short glass fiber rein-forced polybutylene terephthalate (PBT) polymer was investigated by means of cutting several specimens along 0°, 45°, and 90°, in comparison to the injection direction [3]. One of the most interesting aspects is related to the shell-core structure developing in the material during the injection molding process. This fact results in the fiber in the shell region, close to the surfaces of the specimen, being strongly aligned with the polymer flow direction, whereas, in the core of the specimen, they are transversally aligned with the polymer flow. This aspect was also investigated by means of numerical simulations [4,5,6,7,8]. In addition, much effort has also been focused on investigating the influence of the fiber length on the mechanical properties of SFR thermoplastics. Fu and Lauke presented an analytical model for the estimation of the probabilistic fiber length and orientation distribution in injection molded components and validated it by comparing its prediction to experimental results [9]. In their conclusions, they highlighted how the strength of the composite increases with the fiber length as well as with the reduction of the critical fiber length or, in other words, with the increase of the interfacial adhesion between fibers and matrix polymer. Similar conclusions have been also drawn by other researchers [10,11].

Many studies related to the experimental characterization of the mechanical behavior of SFRP under various loading conditions have also been performed [12,13,14,15,16,17,18,19,20,21,22,23,24,25,26,27]. Experiments were conducted by cutting the specimens with various angles from the plates made of glass fiber-reinforced polyamide-6 (PA6) and polyphenylene sulfide (PPS). Changes in mechanical properties according to glass fiber content and fiber orientation angle can be confirmed. Uniaxial tensile and compressive tests for θ = 0°,90° specimens were conducted, and the experimental results were verified with the simulation results and application for complex geometry. Similarly, the stress triaxiality-dependent failure behavior of the polyamide-6 with 20% carbon fiber (PA6-20CF) was investigated [22]. In addition, many studies have been conducted to investigate the mechanical behavior and failure mechanism of polymer composites reinforced with glass fiber and carbon fiber under compressive load [28,29,30,31,32]. The progress of the kink-band and the fracture mode were especially described in detail in terms of fractography [28,29]. Additionally, there were experimental results that confirmed that the mechanical behavior of fiber-reinforced composites can be different depending on the strain rate [6,24,28,29,33,34]. The deviation in stress–strain response was influenced by the interfacial properties because of the strain rate sensitivity of the fiber and matrix. In addition, multi-scale modeling and simulation for characterizing the material mechanical behavior have been utilized [8,35,36,37]. As mentioned earlier, in the cases of composites, since consideration of anisotropy greatly affects the accurate prediction of mechanical properties, attempts to numerically model the fiber orientation through experiments and simulation have been made steadily [38,39,40,41,42,43].

For the mechanical properties of polyamide-6 reinforced with carbon fiber studied in this paper, Karsli and Aytac investigated the thermal and morphological properties [44]. They focused on the influences of fiber length and fiber content. Additionally, the failures of fibers and matrix were analyzed through SEM image analysis of fracture surfaces of the specimens. In addition, Zhang investigated the strain rate and temperature-dependent mechanical properties of carbon fiber-reinforced thermoplastic composites fabricated by hot press molding [45]. This research presented that the modulus and strength of continuous carbon fiber reinforced thermoplastic (CCFRT) increase with a higher strain rate. Additionally, the relationship is non-linear, and it can be confirmed that it is due to the increase in the strength of the thermoplastic matrix and the fiber interface. In this study, changes in mechanical properties of PA6-20CF manufactured through injection molding according to fiber orientation distribution and strain rate are identified. Additionally, by appropriately considering local tensile-compressive mechanical properties caused by injection molding, contributed to the systematization of the structural design of complex structures. The research is articulated according to the following goals. First, experimental characterization of PA6-20CF through specimens of different thicknesses and angles fabricated by injection molding. Second, verification by injection molding simulation. Last, consideration of local mechanical properties and verification of tensile-compressive mechanical properties through the mapping process. Therefore, this paper presents descriptions of the material and the geometry of the specimen used in the experiment to obtain tensile-compressive mechanical properties according to the fiber orientation. Then, the experimental results are discussed and compared in detail with the analysis of the failure mechanism observed in SEM images. Based on the anisotropic elastoplastic mechanical behavior of the material, the numerical model is explained, and simulations were conducted. Considering the strain rate-dependent tensile-compressive mechanical properties, the numerical model coefficients according to the fiber orientation were derived by calibration of the load–displacement results of the experiment and simulation. As a final step, mechanical properties are input based on the obtained coefficients, and a bending test under static and high strain rate conditions was conducted which was machined from a cross member manufactured by injection molding of PA6-20CF (each jig speed: 1 mm/min, 20 mm/min). It allowed evaluation of how much the previously described mechanism can improve the failure prediction accuracy in the analysis of complex geometries. Under the static and high strain rate conditions, failure was predicted with small errors of about 9.6% and 9.3%, respectively. Therefore, the validity of the derived sets of properties was confirmed, suggesting that these research procedures can improve the reliability of the structural analysis of composite materials with complex shapes under complex stress states and contribute to design systematization.

## 2. Materials and Methods

### 2.1. PA6-20CF Plane Specimen

**Figure 1:**
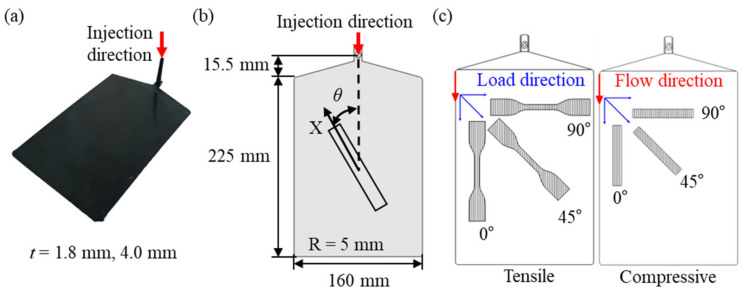
(**a**) PA6-20CF plate manufactured by injection molding, and injection direction, (**b**) Schematic illustration of different fiber orientation (𝜃) specimens obtained from the injection-molded plate (X = loading axis), (**c**) fiber alignment in specimen with fiber orientation (*θ*) = 0°, 45°, 90°.

In this study, PA6-20CF material reinforced with KN111 polyamide-6 (Kolon Plastics Inc., Gimcheon, Republic of Korea) and Torayca T700S (Toray Carbon Fibers America Inc., Tacoma, USA.) short carbon fiber with a weight fraction of 20% was investigated. PA6-20CF plate specimens with a thickness of *t* = 1.8 mm and 4.0 mm were manufactured through the injection molding of the PA6-20CF pellet, and the specifications are shown in Figure 1a, b. Before the injection process of the plate specimen, the pellet was dried at 120 °C for 6 h to prevent deterioration of properties due to hygroscopicity and was injected under the following process conditions: the mold surface temperature of 85 °C, melt temperature of 285 °C, injection time with 2 sec, and cooling time with 20 sec. After that, specimens were manufactured within a small processing error range of 0.25 mm through wire cutting of the plates. Tensile and compression specimens were prepared for three orientation angles *θ* = 0°, 45°, and 90°, which are defined as the angle between flow direction with the direction of applied load during tests (see Figure 1b,c).

**Figure 2:**
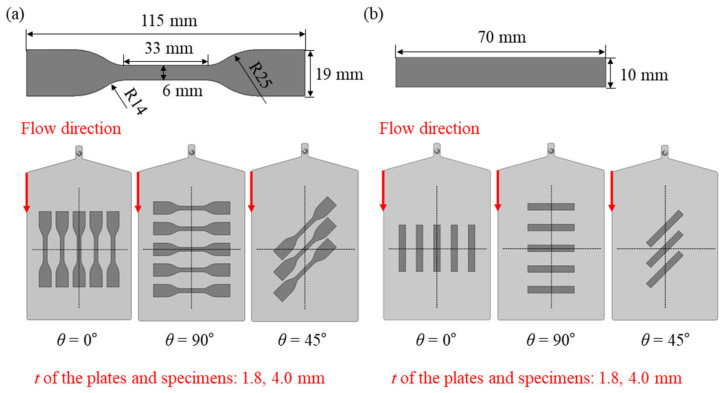
(**a**) ASTM-D 638-02a-Type IV specimen dimensions and cutting location of the tensile specimens from the plate, (**b**) ASTM-D3410M-16 specimen dimensions and cutting location of the compressive specimens from the plate.

In addition to that, the machining locations of the specimen from the plate were decided through finite volume method (FVM) analysis, and a detailed description will be presented in Section 2.2. After wire cutting, all the machined specimens were stored in a sealed plastic bag with silica gel to prevent moisture absorption. To prevent deviations in physical properties due to the hygroscopicity of all specimens, prior to the test, the HKOD-50 electric furnace (heat flow: 12 m^3^/h) was used for drying at 120 °C for 6 h, and then the temperature was lowered for 1 h at room temperature. After that, the tests were conducted. The tensile tests were carried out according to ASTM-D638-02a-Type IV standard and the machined positions on the plate are the same at the thickness t = 1.8 and 4.0 mm of the plate as shown in Figure 2a [46]. An Epsilon 3542 extensometer with a gauge length of 25 mm was used. Additionally, the tests were performed with different jig speeds of 1.5, 15, and 150 mm/min for considering strain rate dependency (strain rates of 0.001, 0.01, and 0.1 s^−1^, respectively).

The compressive tests were carried out according to ASTM D3341M-16 standard and the machined positions on the plate are the same at the thickness *t* = 1.8 and 4.0 mm of the plate as shown in Figure 2b [47]. The tests were performed with different jig speeds of 0.6, 6, and 60 mm/min for considering strain rate dependency (strain rates of 0.001, 0.01, and 0.1 s^−1^, respectively). To prevent buckling that occurs in the specimen when a compressive load is applied, the specimen has a relatively short gauge length compared to the grip part. Additionally, it was confirmed that only acceptable failure characteristic results within the ASTM D3341M-16 standard were derived. For repeatability verification, *θ* = 0°, 90°: 5 times, and *θ* = 45°: 3 times or more were performed according to each test condition (fiber orientation and strain rate) both tensile and compressive.

### 2.2. Numerical Implementation

In the research, three different commercial software were utilized. Autodesk Moldflow Insight (AMI) 2019 (San Rafael, CA, USA) was employed for the estimation of the fiber orientation distributions in the injection-molded plates and specimens. The Autodesk Helius 2019 (San Rafael, CA, USA) allowed for the mapping of the local mechanical properties, as a result of the fiber length and orientation distributions. The mapping was carried out from the results thermoplastic injection molding (TIM) process simulation into the input file of the ABAQUS/Standard structural simulation. Finally, ABAQUS 2017 (Dassault Systemes, Velizy-Villacoublay, France) was employed for the estimation of local stress and strain distribution on specimens.

#### 2.2.1. Thermoplastic Injection Molding Simulation

**Figure 3:**
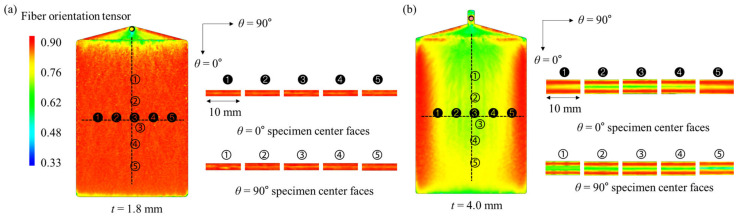
Results of the fiber orientation distribution from cut faces of the specimens from the plate of thickness *t* = (**a**) 1.8 mm, (**b**) 4.0 mm.

The thermoplastic injection molding simulation was developed by means of the commercial software Autodesk Moldflow Insight 2019. To properly locate the specimens on the plate used for machining the tensile and compressive specimens, a finite volume method simulation of injection molding of the plate was conducted as shown in Figure 3. By this action, cross-sections at the center of the specimens could have a consistent fiber orientation distribution.

In the simulation, the same process parameters utilized in the real injection molding process have been utilized, as summarized previously. The fiber orientation distribution was derived through the ARD-RSC model presented in Equation (1) [48,49]. The ARD-RSC model considered the deformation rate tensor ($\dot{\gamma }$) as a polynomial rather than a constant in the original Folgar–Tucker formulation [50]. Thus, the overestimation of the rate of change of the fourth-order fiber orientation tensor was adjusted. As a result, it was improved to better describe the interactions between fibers with a length of 1 mm or longer.

(1) $$\begin{array}{l} \frac{\partial a_{ij}}{\partial t}=-\frac{1}{2}\left(w_{ik}a_{kj}-a_{ik}w_{kj}\right)+\frac{1}{2}\lambda \left({\dot{\gamma }}_{ik}a_{kj}+a_{ik}{\dot{\gamma }}_{kj}-2{\dot{\gamma }}_{kl}\left[a_{ijkl}+\left(1-k\right)\left(L_{ijkl}-M_{ijkl}\cdot a_{mnkl}\right)\right]\right)\\ +\dot{\gamma }\left[2\left(c_{ij}-\left(1-k\right)c_{kl}M_{ijkl}\right)-2k\cdot c_{kk}a_{ij}-5\left(c_{ik}a_{kj}+a_{ik}c_{kj}\right)+10c_{kj}\left(a_{ijkl}+\left(1-k\right)\left(L_{ijkl}-M_{ijkl}\cdot a_{mnkl}\right)\right)\right] \end{array}$$

In Equation (1), the constant *k* is a dynamic scalar factor describing the ability to realign the fibers along the mold after injection. A low *k* means that the orientation ability of the fiber itself is low and, therefore, the degree of agreement between the fiber orientation distribution and the polymer resin flow is low. Conversely, when the value of *k* is large, the fibers are realigned to a high degree of agreement with the direction of resin flow in the mold between injections. In this research for the PA6-20CF, the orientation dynamic scalar factor *k* = 0.5 of ARD-RSC was utilized, which was utilized and verified through the comparison of fiber orientation distribution confirmed by SEM analysis and simulation in previous studies [22,23]. In the TIM simulation, 486,195 and 1,285,994 tetrahedral elements were utilized with *t* = 1.8 mm plates and for *t* = 4.0 mm plates, respectively. The mechanical properties of the PA6-20CF material were inputted in the Moldflow numerical simulation and the constants for the Ramberg–Osgood flow stress model, Equation (2), were calculated and are reported in the next section.

(2) $$\sigma =E^{1/n}{\left(K\right)}^{\left(n-1\right)/n}{\left(\varepsilon_{p,eff}\right)}^{1/n}$$

This procedure allowed mapping the mechanical properties on the injection molding simulation mesh during the filling phase, sparing time during the mapping operation. Along with the flow stress model of Equation (2), a modified Hill ’48 yield function in Equation (3) was utilized. It allowed considering the variation of the mechanical properties, for each element of the mesh, caused by the local fiber length and orientation distributions.

(3) $$\begin{array}{l} \sigma_{eff}=\left[\frac{1}{2}\left\{{\left(\alpha \sigma_{11}-\beta \sigma_{22}\right)}^{2}+{\left(\beta \sigma_{22}-\beta \sigma_{33}\right)}^{2}\right.\right.+{\left(\beta \sigma_{33}-\alpha \sigma_{11}\right)}^{2}\\ {\left.\left.+6\left[{\left(\sigma_{12}\right)}^{2}+{\left(\sigma_{23}\right)}^{2}+{\left(\sigma_{31}\right)}^{2}\right]\right\}\right]}^{1/2} \end{array}$$

The *α* and *β* parameters in Equation (3) account for the improvement of the polymer matrix mechanical properties due to the presence of the carbon fibers, along the direction parallel and transversal to the local flow direction, respectively, and were calculated by Equation (4).

(4) $$\begin{array}{l} \alpha \left(\lambda_{I}\right)=\theta +\left[\frac{\left(\alpha_{m}-\theta \right)}{\left(\lambda_{m,I}-1/2\right)}\right]\left(\lambda_{I}-1/2\right)\\ \beta \left(\lambda_{I}\right)=\theta +\left[\frac{\left(\beta_{m}-\theta \right)}{\left(\lambda_{m,I}-1/2\right)}\right]\left(\lambda_{I}-1/2\right) \end{array}$$

For each element of the mesh, *α* and *β* are calculated based on *λ*_I_, *α*_m_, *β*_m_ and *λ*_m, I_ constants. The *λ*_I_ defines the first eigenvalue of the fiber orientation tensor of the considered element, and the *λ*_m, I_ define the first eigenvalue of the fiber orientation tensor in strong fiber alignment along the flow direction region.

#### 2.2.2. Mapping Procedure

The mapping procedure was carried out in the Autodesk Helius software and allowed us to map the local mechanical properties, as a result of the TIM process, into the ABAQUS numerical simulation. This procedure is an important task in determining how the original fiber orientation distribution is reflected in the specimen when the specimen was machined at each location on the plate. After the mapping operation, the ABAQUS input file containing a specific set of mechanical properties, for each element of the mesh, was created and ought to be run from the Autodesk Helius command shell.

**Figure 4:**
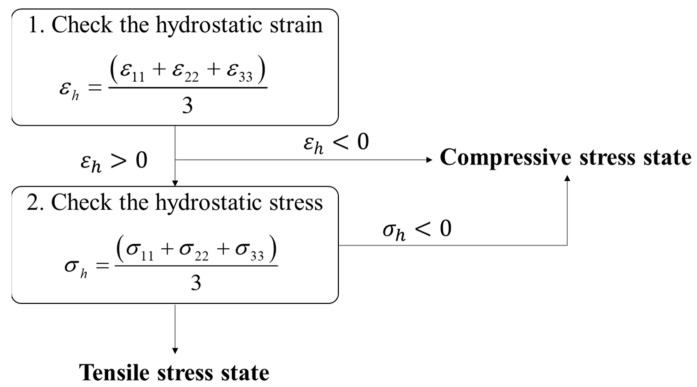
Schematic diagram of the procedure how to define the stress state.

The Ramberg–Osgood model constants are based on isotropic behavior, and the respective σ-ε curves for tension and compression were considered. Once the stress state is determined, the program inputs the mechanical properties suitable for the stress state of each element. At each iteration step, both tensile and compressive solutions are calculated using a set of tensile and compressive mechanical properties. Then, the stress state is determined according to two verification procedures as shown in Figure 4, and a corresponding appropriate solution is used for each step.

#### 2.2.3. Structural Simulation

The structural numerical simulations were constructed into ABAQUS/Standard utilizing the C3D10—quadratic hexahedral elements. Both tension and compression specimen geometries were modeled identically to the specifications of the specimen used in the test as previously shown in Figure 2. The region connected to the jig was given a rigid body condition, and in the case of the tensile specimen, displacement at the gauge distance node was used as output data. For *t* = 1.8 mm specimens, 9216, 22,400 elements were used for tensile and compressive specimens. Additionally, for *t* = 4.0 mm specimens, 18,432, 40,800 elements were used, respectively. The results of the ABAQUS numerical simulations are reported in detail in Section 3.3 and Section 3.4 of the paper.

## 3. Results and Discussions

### 3.1. Experimental Results

In this section, the results of tensile and compressive tests performed at room temperature for specimen thicknesses *t* = 1.8, 4.0 mm, fiber orientation angles *θ* = 0°, 45°, 90°, and strain rates 0.001, 0.01, and 0.1 s^−1^ are presented. Furthermore, the mechanical properties and the behavior of the anisotropic elastoplastic material derived for each test condition are discussed. Afterward, it was analyzed based on the fractographic study of the SEM image, which is discussed in detail in Section 3.2.

#### 3.1.1. Tensile Tests

**Figure 5:**
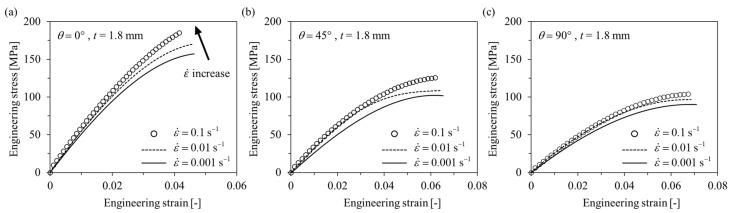
Tensile engineering stress–engineering strain curves of *t* = 1.8 mm specimens with different strain rates. (**a**) *θ* = 0°, (**b**) *θ* = 45°, (**c**) *θ* = 90°.

**Figure 6:**
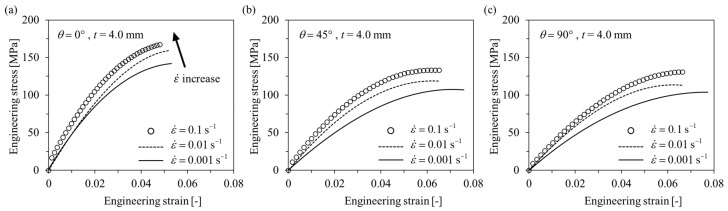
Tensile engineering stress–engineering strain curves of *t* = 4.0 mm specimens with different strain rates. (**a**) *θ* = 0°, (**b**) *θ* = 45°, (**c**) *θ* = 90°.

Engineering stress-engineering strain curves obtained as a result of the tensile tests are shown in Figure 5 and Figure 6. Additionally, the anisotropic elastoplastic material behavior (with hardening) of short fiber reinforced polymer (SFRP) according to the strain rate can be observed. As the fiber orientation angle *θ* decreased and strain rate increased at *t* = 1.8 mm and 4.0 mm, ultimate tensile strength *σ*_UTS_, elastic modulus, and yield strength tended to increase. On the other hand, ultimate tensile strain *ε*_UTS_ tended to decrease as the fiber orientation angle *θ* decreased and the strain rate increased. Based on this, it shows that the material became brittle as the strain rate increased. When the thickness *t* of the plate is different (*t* = 1.8 and 4.0 mm), different material behavior is shown because of the deviation in the degree of anisotropy. For *θ* = 0°, the strength is greater at *t* = 1.8 mm, but for *θ* = 45° and 90°, the strength is greater at *t* = 4.0 mm. This is because the thinner the plate, the higher the shell ratio in the shell-core structure (higher average fiber orientation index), and the better the fibers are aligned in the main flow direction (Figure 3). Additionally, for the same reason, when *t* = 1.8 mm, it shows big differences in mechanical properties between *θ* = 0° and *θ* = 45°, 90°, but small at *t* = 4.0 mm.

#### 3.1.2. Compressive Tests

**Figure 7:**
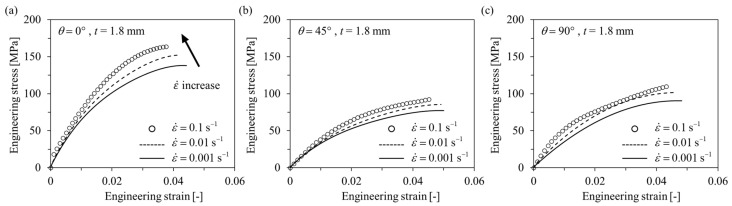
Compressive engineering stress–engineering strain curves of *t* = 1.8 mm specimens with different strain rates. (**a**) *θ* = 0°, (**b**) *θ* = 45°, (**c**) *θ* = 90°.

**Figure 8:**
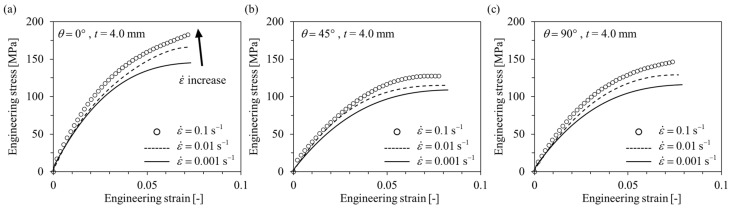
Compressive engineering stress–engineering strain curves of *t* = 4.0 mm specimens with different strain rates. (**a**) *θ* = 0°, (**b**) *θ* = 45°, (**c**) *θ* = 90°.

Engineering stress-engineering strain curves obtained as a result of the compressive tests are shown below in Figure 7 and Figure 8. Similar to the tensile test results, the behavior of the anisotropic elastoplastic material of the short fiber reinforced polymer according to the strain rate could be observed, and different results were obtained when the thickness *t* was changed. At *t* = 1.8 mm, the strain rate sensitivity of *θ* = 0° is greater than that of *θ* = 45°, 90°. At both *t* = 1.8 mm and 4.0 mm, the ultimate compressive strength *σ*_UCS_, elastic modulus, and yield strength tended to increase as the strain rate increased. On the other hand, the ultimate compressive strain *ε*_UCS_ tended to decrease as the strain rate increased.

Comparing the results of the compressive test with the tensile test, it can be observed that the short fiber-reinforced polymer exhibited completely different mechanical behavior under tensile and compressive loading conditions. First, under the compressive load condition at the same strain rate, it showed that the *σ*_UCS_ at *θ* = 45° was the lowest and the *ε*_UCS_ was the biggest. In addition, it can be observed that the difference in *σ*_UTS_ between *t* = 1.8 mm and *t* = 4.0 mm was relatively smaller than the difference in σ_UCS_. Furthermore, in compression, when *t* = 4.0 mm, it showed that the strength was higher than that of *t* = 1.8 mm, at all *θ*. From this, it can be concluded that the mechanical properties of the short-fiber reinforced polymer are relatively strongly influenced by the matrix in the compressive state [31,32,45].

### 3.2. SEM Image Analysis

In this section, the experimental results explained in Section 3.1 are analyzed and verified through SEM (Scanning Electron Microscope) image analysis of the fracture surface of the specimen. For SEM analysis, a JSM-7100F FE-SEM machine (JEOL Ltd., Tokyo, Japan) with a resolution of 3.0 mm (at 15.0 kV) was utilized. The specimen fracture faces were coated with Pt, and SEM imaging was taken perpendicular to the fracture surface. The fiber orientation at the fracture surface and failure characteristics of the matrix and fiber were analyzed.

#### 3.2.1. Tensile Specimens

**Figure 9:**
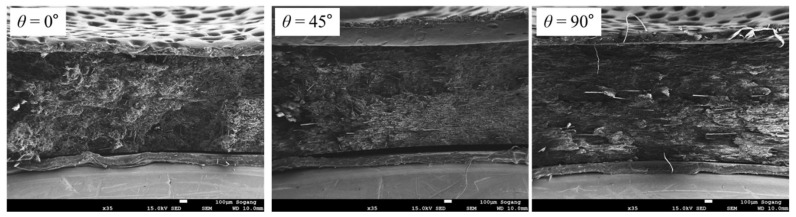
Fracture faces observed from tensile specimens which were tested with a strain rate of 0.001 s^−1^ (magnification: ×35, scale: 100 µm).

Through SEM analysis of the tensile specimen, it can be confirmed that the alignments of the fibers observed at the fracture surface were different depending on the fiber orientation *θ* (see Figure 9). It can be observed that the fibers of the *θ* = 0° specimen are cut perpendicular to the fracture plane, and in the case of the *θ* = 45° and 90° specimens, the fibers are aligned parallel to the fracture plane. In addition to that, the length of the fibers observed at the fracture surface of the *θ* = 90° specimen is the longest, and the length of *θ* = 0° is the shortest [3,21].

**Figure 10:**
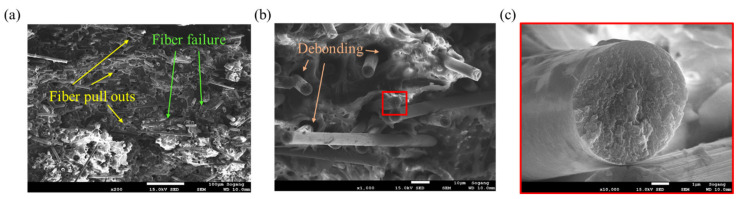
Fracture mechanisms observed for *θ* = 0° tensile specimen: (**a**) Fiber failure and fiber pull-outs (magnification: ×200, scale: 100 µm), (**b**) fiber-matrix debonding (magnification: ×1000, scale: 10 µm), (**c**) fracture face of the fiber after the tensile failure (magnification: ×10,000, scale: 1 µm).

In the case of *θ* = 0°, since the fracture plane is perpendicular to the fiber direction, it can be expected that the fiber fracture occurs predominantly. Various microscopic damage mechanisms according to the fiber orientation *θ* under tensile load can be observed. It is possible to observe fiber failure of shortened fibers after breakage and fiber pull-outs in which fibers were pulled out from the matrix in Figure 10a. Additionally, as shown in Figure 10b, debonding occurred as traces in which fiber separated from the matrix [51]. The fracture face of the fiber in Figure 10c is consistent with the fracture characteristics of the carbon fiber under the tensile load studied previously [52,53,54].

**Figure 11:**
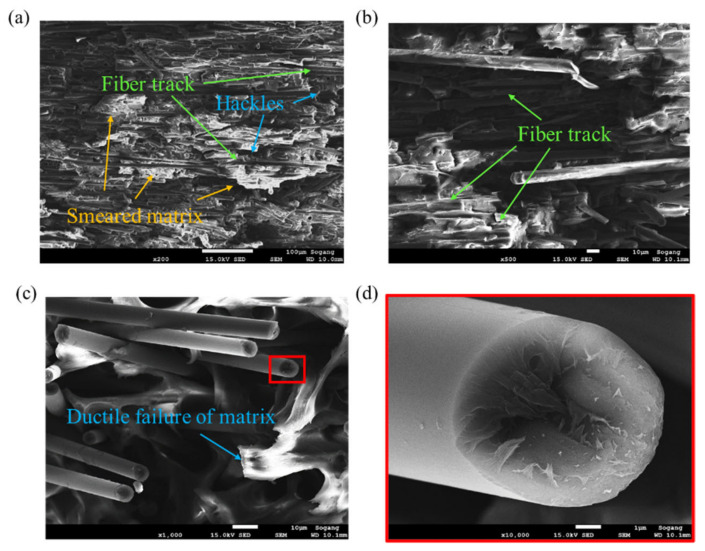
Fracture mechanisms observed for *θ* = 90° tensile specimen: (**a**) Fiber tracks, hackles, and smeared matrix (magnification: ×200, scale: 100 µm), (**b**) fiber tracks and broken long fiber (magnification: ×500, scale: 10 µm), (**c**) ductile failure of the matrix (magnification: ×1000, scale: 10 µm), (**d**) smooth fracture face of the fiber (magnification: ×10,000, scale: 1 µm).

**Figure 12:**
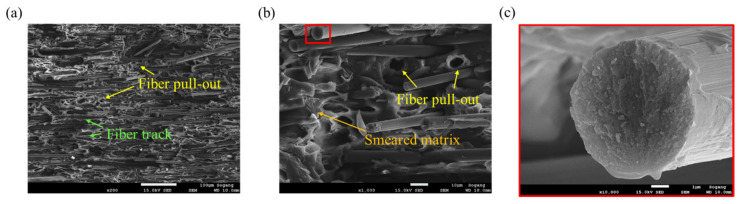
Fracture mechanism observed for *θ* = 45° tensile specimen, (**a**) fiber pull-outs and fiber tracks (magnification: ×200, scale: 100 µm), (**b**) smeared matrix and fiber pull-outs (magnification: ×1000, scale: 10 µm), (**c**) fracture surface of the fiber (magnification: ×10,000, scale: 1 µm).

At the failure surface of the *θ* = 90° specimen, completely different failure characteristics were observed, as shown in Figure 11. The average length of the fibers was long. Additionally, the fiber tracks left as traces after the fibers were separated from the matrix and the hackles distributed along them can be observed (Figure 11a,b). Moreover, the matrix that was bonded to the fibers was weakened and a smeared matrix appeared. Through the deformation of the matrix, the ductile behavior of *θ* = 90° can be explained. In the case of *θ* = 45°, the behavior is intermediate between *θ* = 0° and *θ* = 90° (Figure 12). Although the average fiber length observed was long, a large number of fibers in which breakage has occurred can also be found. Therefore, it can be confirmed that the failure characteristics that can be seen at *θ* = 0° and 90° coexist. The fracture face of the fiber was the roughest at *θ* = 0°, and conversely, the smoothest at *θ* = 90°.

#### 3.2.2. Compressive Specimens

**Figure 13:**
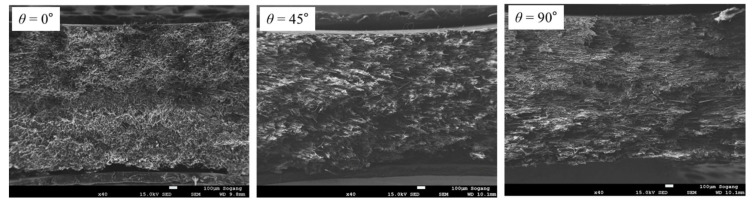
Fracture surfaces observed for compressive specimens with a strain rate of 0.001 s^−1^ (magnification: ×40, scale: 100 µm).

In compressive specimen SEM images in Figure 13, the direction of fibers aligned at each fiber orientation *θ* show similar results with tensile (see Figure 9). However, the average length of fibers observed at the fracture surface did not show a significant difference between *θ* = 45° and *θ* = 90°. Moreover, the deformed matrix appears brighter at *θ* = 45° than at *θ* = 90°. Through this, more deformation of the matrix can be grasped at *θ* = 45°.

**Figure 14:**
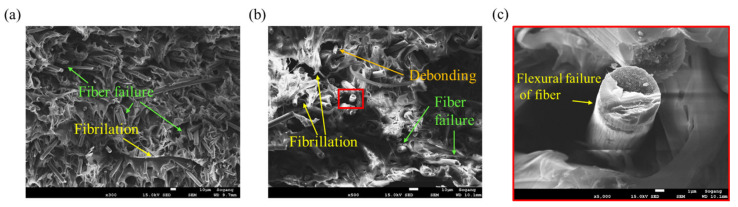
Fracture mechanism observed for *θ* = 0° compressive specimen, (**a**) fiber failure and fibrillation (magnification: ×300, scale: 10 µm), (**b**) fiber-matrix debonding (magnification: ×500, scale: 10 µm), (**c**) fracture face of the fiber after flexural failure (magnification: ×5000, scale: 1 µm).

**Figure 15:**
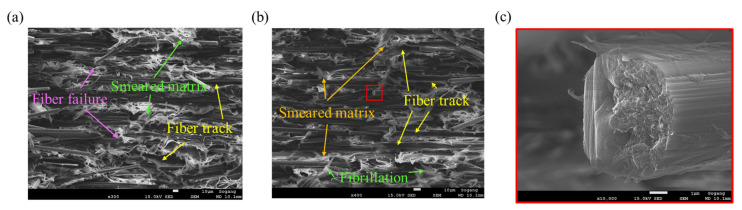
Fracture mechanisms observed for *θ* = 90° compressive specimen: (**a**) fiber failure, smeared matrix, and fiber tracks (magnification: ×300, scale: 10 µm), (**b**) fiber tracks and fibrillation (magnification: ×400, scale: 10 µm), (**c**) fracture face of the fiber after flexural failure (magnification: ×10,000, scale: 1 µm).

For *θ* = 0° compressive specimen, the failure characteristics were similar to those of the tensile specimen, but in compression, fiber breakage was promoted by the buckling of the fiber, and fibrillation induced by strong shear ductile damage occurred, as shown in Figure 14a,b. Additionally, fiber-matrix debonding can be observed in Figure 14b,c [51]. The fracture surface of the fiber in Figure 15c is comparable to the fracture characteristics of the carbon fiber under flexural load studied previously [52,53].

**Figure 16:**
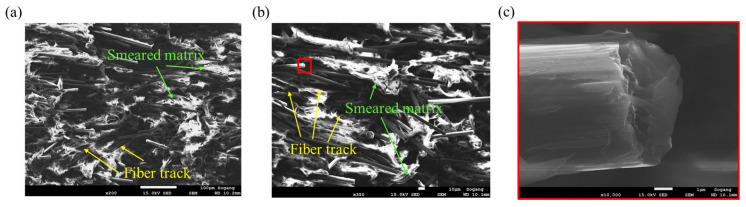
Fracture mechanism observed for *θ* = 45° compressive specimen: (**a**) smeared matrix and fiber tracks (magnification: ×200, scale: 100 µm), (**b**) smeared matrix and fiber tracks (magnification: ×350, scale: 10 µm), (**c**) fracture surface of fiber due to flexural failure (magnification: ×10,000, scale: 1 µm).

Both in *θ* = 90° and 45°, fiber tracks, the smeared matrix near the fiber, and fibrillation occurred frequently as shown in Figure 15 and Figure 16. In compression, fibers commonly showed flexural failure due to buckling, and ductile shear damage was dominant [28,29,30,31,32,52,53,54]. Fiber failure was observed more frequently at *θ* = 90° than at *θ* = 45°, conversely, there was more deformation on the matrix in the case of *θ* = 45°. These facts support that the σ_UCS, 90°_, is bigger than σ_UCS, 45°_.

### 3.3. Numerical Model Verification

**Figure 17:**
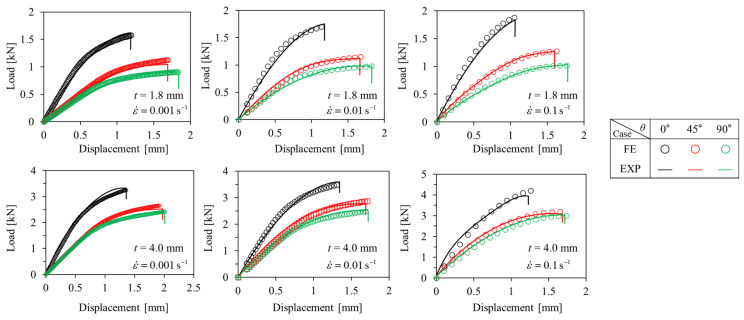
Comparison between experimental and numerical results for ASTM-D638 tensile specimens.

**Figure 18:**
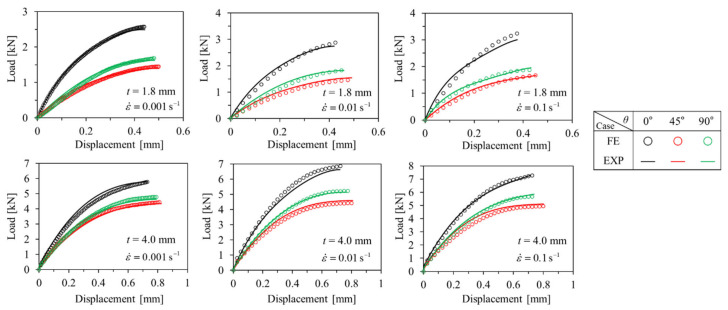
Comparison between experimental and numerical results for ASTM-D3410 compressive specimens.

To prove the reliability of the mapping procedure and the anisotropic elastoplastic behavior of PA6-20CF, EXP-FE load–displacement curves for tensile and compression specimens were compared. The same Ramberg–Osgood coefficients were applied to both *t* = 1.8 and 4.0 mm at the same load condition and strain rate. Additionally, the comparison result of the load–displacement curves of the EXP-FE under each test condition (load condition and strain rate) is shown in Figure 17 and Figure 18. The input coefficient sets were as shown in Table 1. In both tension and compression, as the strain rate increased, *K*, *n* decreased, *α*, *β*, and the elastic modulus of matrix and fiber tended to increase. Moreover, in the case of compression, the change in the elastic modulus was more significant. As mentioned earlier, different fiber orientation distribution causes a difference in mechanical properties, and it is important to consider this properly. By the mapping procedure, local mechanical properties as a result of the injection molding process can be input for each element in FE simulation, so the reliability of the results can be improved. The possibility to consider the thickness-dependent (fiber orientation-dependent) mechanical properties with one set of model coefficients is a huge advantage for the simulation of complex geometries. The coefficients derived from the EXP-FE comparison were finally verified by being used for structural analysis of structures with complex shapes under a complex stress state in the next section—Section 3.4.

### 3.4. Application: Composite Cross Member Rear Part Bending Specimen

**Figure 19:**
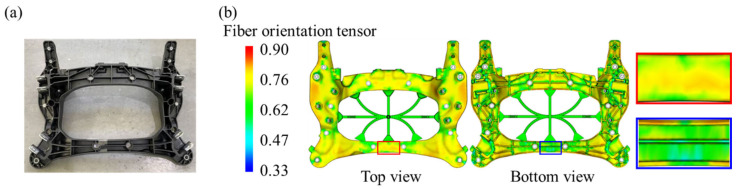
(**a**) PA6-20CF composite cross-member with aluminum insert fabricated by injection molding (bottom view), (**b**) fiber orientation distribution result of the cross member and detailed view at the machined location.

**Figure 20:**
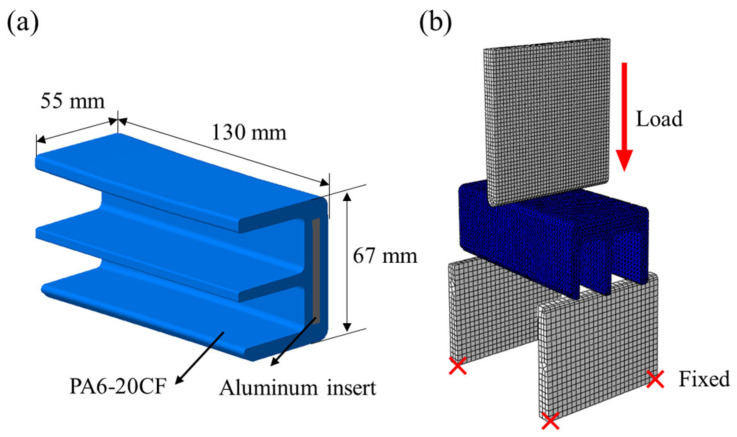
(**a**) Dimensions and configuration of the specimen after cutting, (**b**) FE model and boundary conditions.

In order to verify the effect of considering the tensile-compressive mechanical behavior of PA6-20CF according to the fiber orientation and load conditions, a bending test of a specimen machined from PA6-20CF composite cross member was conducted (see Figure 19 and Figure 20). The cross member was manufactured by injection molding under the following process conditions (Table 2), and TIM analysis was performed by inputting the same process parameters in the simulation. In addition to that, the model coefficients set shown in Table 1 was input to evaluate the reliability of the derived PA6-20CF mechanical properties. By the mapping procedure, the local mechanical properties due to the injection molding were considered in the specimen machined by cutting the cross member. In order to secure the reliability of the mechanical properties at the static and high strain rates derived previously, the jig speed was varied, and EXP-FE comparison was performed for two cases (jig speed: 1 mm/min, 20 mm/min). The experiment setup is the same as shown in Figure 20, and for the bending experiments, a universal test-one machine was used. 

**Figure 21:**
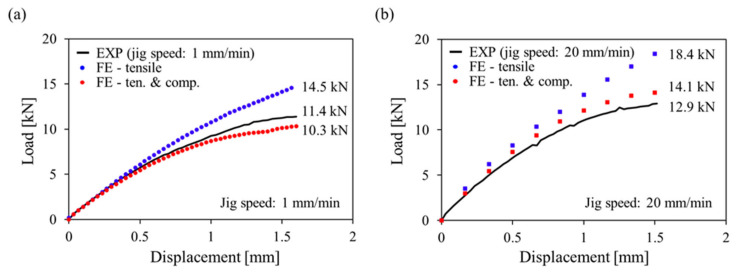
Bending test results comparison between EXP and FE with jig speed: (**a**) 1 mm/min, (**b**) 20 mm/min.

In Figure 21, the bending test load–displacement curves for two different jig speeds were compared. The simulation results in which only tensile properties were input and results in which both tensile and compressive properties were entered were compared with the experimental results. In the case of the jig speed with 1 mm/min, as seen in Figure 21a, when only the tensile properties were considered, the result was 3.1 kN higher than the experimental result (relative error: 27.2%), whereas when tensile and compressive properties were simultaneously considered, the result was 1.1 kN lower (relative error: 9.6%). In addition to that, the result of the jig speed with 20 mm/min is shown in Figure 21b. When only the tensile properties were input, there was an error of 5.5 kN (relative error: 42.6%), and when both were considered, an error of 1.2 kN was obtained (relative error: 9.3%). By comparing the load at the failure displacement, it can be seen that the error was improved by about 26% at the jig speed of 1 mm/min and by about 13% at 20 mm/min. In the case of PA6-20CF, the tensile and compressive mechanical behaviors are different due to carbon fibers, and this reinforces the argument that these should be properly considered. Additionally, comparing Figure 21a,b, it can be confirmed that the prediction error was significantly reduced when the strain rate was properly considered. As a result, these show that investigated procedures can contribute to predicting the failure of the PA6-20CF structures. 

## 4. Conclusions

The main objectives of this paper were as follows: first, failure mechanism investigation through experimental characterization and SEM verification of injection molded PA6-20CF; second, defining the advantage of simultaneous consideration of tensile-compression properties of anisotropic elastoplastic material behavior in the complex stress state; third, design systemization of SFRP which is fabricated by injection molding considering mechanical properties according to fiber orientation distribution and strain rate.

By experiments and SEM image analysis, mechanical behavior of PA6-20CF with various load conditions and fiber orientation was identified. Additionally, it was found that the failure mechanism was very different depending on the fiber orientation and the loading conditions. Afterwards, utilizing the mapping procedure, inputting local mechanical properties induced by the injection molding process into the structural numerical simulation was carried out. By this method, it was possible to accurately consider the anisotropic mechanical behavior of the uniaxial tensile and compressive specimen, and by the comparison of EXP-FE results, the Ramberg–Osgood flow stress model coefficients were derived. A structural analysis model was constructed in which the mechanical properties at each load condition and strain rate were inputted and, to verify it, a bending test was conducted with a specimen manufactured by machining a composite crossmember. It demonstrated high EXP-FE correlation under static conditions and high strain rate conditions to prove the validity and applicability of modeling.

## Figures and Tables

**Table 1:** Model constants for the Ramberg–Osgood flow stress model and for the modified Hill’48 yield function.

Stress State	Strain Rate [s^−1^]	*K* [MPa]	*n*	*E*_m_ [GPa]	*E*_f_ [GPa]	*α*	*β*	*λ*_i_
Tension	0.001	83.4	10.4	1.3	50.1	2.795	1.574	0.85
	0.01	79.3	9.9	1.4	55.1	2.929	1.606	0.85
	0.1	77.9	9.2	2.1	63.7	3.031	1.612	0.85
Compression	0.001	42.6	10.6	1.6	50.7	1.662	0.435	0.85
	0.01	40.6	9.1	3.7	65.9	1.811	0.575	0.85
	0.1	38.2	8.8	4.0	70.2	2.222	0.613	0.85

**Table 2:** Injection molding process parameters of PA6-20CF composite cross member.

Process Parameter	Value
Mold surface temperature [°C]	85
Melt temperature [°C]	285
Injection time [s]	20
Cooling time [s]	140

## Data Availability

The data presented in this study are available on request from the corresponding author.
